# Opportunities and Challenges for Promoting Sustainable Diets within “Food is Medicine” Programs in the United States: A Qualitative Research Study

**DOI:** 10.1016/j.cdnut.2026.109541

**Published:** 2026-09-23

**Authors:** Katherine Consavage Stanley, Paige B Harrigan, Bailey Houghtaling, Elena Serrano, Adrienne Holz, Valisa E Hedrick, Vivica I Kraak

**Affiliations:** 1 World Food Policy Center, Duke University, Durham, NC, United States; 2 Department of Human Nutrition, Foods, and Exercise, Virginia Polytechnic Institute and State University (Virginia Tech), Blacksburg, VA, United States; 3 Center for Nutrition & Health Impact, Omaha, NE, United States; 4 Virginia Family Nutrition Program, Virginia Tech, Blacksburg, VA, United States; 5 School of Communication, Virginia Tech, Blacksburg, VA, United States

**Keywords:** Food is Medicine, Food as Medicine, sustainable diets, plant-rich dietary patterns, qualitative research, planetary health, eating practices

## Abstract

**Background:**

The United States population is experiencing concurrent, interrelated challenges of diet-related chronic diseases, food and nutrition insecurity, and climate change. Food is Medicine (FIM) has been described as a range of food-based programs that aim to prevent, manage, and treat diet-related chronic diseases in partnership with health care systems. Research is lacking in how these programs could promote plant-rich dietary patterns and other sustainability practices to support both human and planetary health.

**Objectives:**

This study explored how FIM experts in the United States define FIM and plant-rich dietary patterns and their views on the opportunities and challenges to incorporating sustainable diet practices into FIM programs.

**Methods:**

We conducted semistructured Zoom interviews with 20 national FIM experts between July and October 2024. Participants were recruited through purposive and snowball sampling. Data were analyzed using inductive and deductive thematic analysis guided by the socioecological model.

**Results:**

Expert perspectives differed on the definition and scope of FIM but largely agreed on the components of a plant-rich dietary pattern. Participants viewed produce prescriptions and medically tailored meal and grocery programs as aligned with plant-rich dietary patterns but described challenges with the lack of standardization across programs. Experts described pilot FIM programs that have incorporated sustainability practices but lacked explicit messaging on the human and planetary health cobenefits. Supporting local or regenerative producers and reducing food waste were identified as sustainability practices that could be systematically incorporated into FIM programs. Challenges included misalignment with the political environment and policy and systems barriers that restrict enabling environments for supporting sustainable diets.

**Conclusions:**

The FIM movement offers promising opportunities to promote collaboration among food and health system actors, but challenges require coordinated actions within and across sectors. Future FIM programs could identify potential areas of human and planetary health cobenefit and incorporate sustainable diet principles into program design and evaluation.

## Introduction

Persistent diet-related chronic disease, food and nutrition insecurity, and climate change collectively threaten human health, the environment, and livelihoods in the United States [1]. Obesity, type 2 diabetes, cardiovascular disease, and other diet-related chronic diseases are the leading causes of death and disability among the United States population [[2], [3], [4]]. Addressing these concurrent challenges requires that food and health systems stakeholders integrate cross-cutting policies, systems, and environmental changes to improve planetary health—defined as the combined health of human civilization and the natural systems on which humans depend [[5], [6], [7]]. This includes shifting toward plant-rich dietary patterns that support human health and well-being and minimize adverse environmental impacts [1,[8], [9], [10], [11]]. These patterns emphasize the intake of high-quality, minimally processed plant-based foods and encourage moderate to minimal intake of animal-based food products, particularly red and processed meats [8,11,12]. Sustainable diets can promote local culture and foods, reduce food loss and waste, preserve biodiversity, and minimize adverse food system impacts on greenhouse gas emissions, water, and land use, among other principles [11].

Food is Medicine (FIM)—a range of food-based interventions that aim to prevent, manage, and treat diet-related chronic diseases in partnership with healthcare systems [[13], [14], [15]]—could offer an opportunity to promote plant-rich dietary patterns and other sustainable diet principles in food and health systems innovations. The range of FIM programs has been widely described using the FIM Pyramid [16,17]. The FIM Pyramid depicts prevention-level programs at the bottom (population-level healthy food policies and programs and government nutrition security programs), followed by more intensive prevention and treatment programs (produce prescription programs and then medically tailored grocery [MTG] programs). Medically tailored meal (MTM) programs are at the top of the Pyramid as the most intensive, treatment-based program [16,17]. Nutrition education and counseling are depicted as cross-cutting components of the top 4 FIM Pyramid levels (see Mozaffarian et al. [16] for a visual depiction of the FIM Pyramid). The 2022 White House Conference on Hunger, Nutrition, and Health and former National Strategy on Hunger, Nutrition, and Health increased stakeholder and public attention and federal, state, and local investments in FIM programs [18,19]. Likewise, in recent years, states have increasingly utilized Medicaid Section 1115 demonstration waivers to provide Medicaid-covered pilot FIM programs that aim to improve diet quality and reduce diet-related chronic diseases [20].

Although the focus of FIM to date has been on how MTM, MTG, and produce prescription programs can reduce food and nutrition insecurity and improve diet-related chronic disease outcomes [13,15,21], some individuals and groups have also described the potential for FIM programs to support sustainable diets and planetary health [[22], [23], [24]]. Bernstein and Hunnes [22] mentioned encouraging individuals and populations to adopt plant-based diets and reducing food waste as practices that FIM programs could adopt to mitigate the health and food systems’ contributions to climate change. However, these authors did not describe how FIM programs could incorporate these practices in the United States or the associated challenges with doing so. This study sought to address this research gap by exploring how United States experts defined FIM and plant-rich dietary patterns and the challenges and opportunities to incorporating sustainable diet practices within FIM programs to support human and planetary health.

## Methods

This study used codebook thematic analysis [25], guided by a qualitative description methodological orientation approach, to identify emergent themes from open-ended, semistructured interviews. The Virginia Tech Institutional Review Board exempted this study from human subject review in June 2024. Study reporting followed the Consolidated Criteria for Reporting Qualitative Research checklist to enhance transferability and confirmability [26].

### Participant identification and recruitment

The research team used stakeholder mapping [27] to identify relevant US FIM experts, organizations, and other entities involved in FIM research, implementation, and dissemination at the national level. The lead investigator (KCS) searched Google and Google Scholar in March 2024 using keywords such as “Food is Medicine” and “Food as Medicine” to identify peer-reviewed and gray literature sources, including FIM-related conference websites, webinars, and journal articles. We prioritized sources published online leading up to and following the 2022 White House Conference and the National Strategy on Hunger, Nutrition, and Health given the increased national investment and attention to FIM that resulted from these events [19]. The search identified individuals from 50 organizations or entities that worked on FIM at a national level (compared with state or community levels), which were tracked within an Excel Sheet. The research team then categorized these entities into 7 groups: *1*) nonprofit public interest, research, and private philanthropic organizations; *2*) academia; *3*) government; *4*) industry, private sector firms, or business-interest organizations; *5*) lobbying or advocacy groups; *6*) professional societies; and *7*) digital news and media.

Stakeholder mapping informed a combined purposive and snowball sampling strategy to identify potential participants. From the stakeholder mapping, researchers identified 3–5 individuals from each group to contact, prioritizing those whose contact information was publicly available. Participants were recruited via e-mail or LinkedIn. After each interview, the research team asked participants who else they thought should be interviewed for this study. Researchers triangulated these data with the stakeholder mapping with the goal of interviewing ≥2 individuals representing each stakeholder group to collect a diverse range of perspectives.

The study aimed to recruit 20 participants following recommendations for achieving data saturation from qualitative interviews [[28], [29], [30]]. Through purposive sampling, we contacted 55 individuals from 44 organizations or entities for interviews; 16 accepted, 10 declined, and 29 did not respond. Snowball sampling led to contacting 8 more individuals; 4 accepted, 2 declined, and 2 did not respond.

### Data collection

The lead researcher (KCS) conducted 20 semistructured one-on-one Zoom interviews between July and October 2024. The interviewer, a White female master’s degree-holding PhD candidate at the time of interviewing, was trained in qualitative research analysis. Co-researchers with qualitative expertise provided input throughout the study, including giving feedback on the draft interview guide and supporting codebook refinement. Interviewees did not have any direct working relationships with the interviewer.

Before the scheduled interviews, the researchers asked participants to complete a short demographic survey and electronic consent via QuestionPro. Researchers shared an abridged version of the 10-item semistructured interview guide with participants in advance to support participant reflexivity and to foster deeper exploration of the study topic. The full interview guide is included in Supplemental File 1. The abridged version sent to participants omitted any discussion of the study’s definitions for sustainable diets or plant-rich dietary patterns to allow participants to explore their own definitions for these terms. The lead investigator (KCS) pilot tested the semistructured interview guide with 2 individuals familiar with food system sustainability and FIM scholarship, from which the guide was further refined.

Before starting each interview, the interviewer restated the key points from the consent form and asked them to provide consent to be recorded. Interview questions explored how participants defined FIM and plant-rich dietary patterns. After participants shared their definitions, the interviewer shared the sustainable diet and plant-rich dietary pattern definitions and principles used to inform this study [11]. Given the direct connection the research team saw between FIM programs and plant-rich diets, we used plant-rich dietary patterns as an entry point through which to explore participant conceptions of other sustainable diet practices and their applicability to FIM programs. KCS used the FIM Pyramid as a visual guide to help participants self-select and share which FIM interventions they had the most experience with. Subsequent questions focused on whether FIM programs incorporated sustainable diet practices and the opportunities and challenges with doing so. KCS adapted the interview questions based on participants’ self-reported experience and utilized probing and follow-up questions as relevant. The researcher also asked participants about the benefits and challenges of publicly positioning FIM as supportive of human and planetary health. The research team did not provide financial compensation to study participants. The interviewer listened to the recorded interviews and corrected the autogenerated Zoom transcripts as needed to ensure accuracy and to add nonverbal cues and other relevant contextual elements.

### Data analysis

Researchers used codebook thematic analysis to analyze the interview data [25,31]. An iterative inductive–deductive approach to coding and codebook development was aided by NVivo version 15. Two researchers, KCS and BH, independently coded 5 transcripts, from which they developed an initial codebook [32]. The researchers met to address disagreements in coding, discuss emerging themes, and refine the codebook. Because emerging themes closely aligned with the levels of the socioecological model (SEM), researchers decided on this deductive structure to organize the concepts identified inductively. KCS and PH then applied the codebook to the remaining 15 interviews while remaining open to the addition, refinement, or collapse of codes throughout the data review process. The 2 researchers independently coded each transcript and met to resolve disagreements and achieve consensus, with consensus decisions and changes to the codebook documented to support dependability in the study approach. For this analysis, researchers defined FIM as the top 3 levels of the FIM Pyramid (MTM, MTG, and produce prescription programs), whereas the bottom 2 levels (nutrition security programs and population-level healthy food policies and programs) were included in the “Policy Environment” SEM level. This definition was derived inductively based on researchers’ reflections on the emerging codes and themes.

Saturation was defined as no emergence of new subthemes, which occurred after 17 interviews [29]. The 3 remaining transcripts were coded, and no new subthemes emerged, reinforcing data saturation. Participants were not asked to provide feedback on the codebook iterations or study findings.

## Results

The 20 interviewees spanned 7 expert groups (Table 1). Seventy percent were women, and most (90%) participants held a master’s or doctoral degree. Participant-reported ages and races or ethnicities varied, with most participants aged between 35 and 49 y and White (Table 2).

**TABLE 1:** Study participants (*n* = 20) categorized by FIM expert group

FIM expert group^1^	*n* (%)	Corresponding participant IDs
Nonprofit public interest, research organization, or private philanthropic organization	11 (36.66)	P1, P4, P5, P7, P10-12, P16-18, P19
Academia	7 (23.33)	P2, P4, P7, P12-14, P16
Government	5 (16.67)	P3, P6, P15, P18, P20
Industry, private sector firm, or business-interest organization	2 (6.67)	P9, P18
Lobbying or advocacy	2 (6.67)	P17, P18
Professional society	2 (6.67)	P12, P18
Digital news and media	1 (3.33)	P8

Abbreviation: FIM, Food is Medicine.

1 Participants could self-identify with >1 expert group.

**TABLE 2:** Sociodemographic characteristics of the Food is Medicine expert participants (*n* = 20)

Characteristic	*n* (%)
Gender	
Woman	14 (70)
Man	6 (30)
Age group (y)	
18–34	1 (5)
35–49	13 (65)
50–64	5 (25)
65–80	1 (5)
Race or ethnicity^1^	
White	15 (68.18)
Hispanic, Latino/a, or Spanish descent	1 (4.55)
Black or African American	3 (13.64)
Asian or Pacific Islander	1 (4.55)
Native American or Indigenous American	2 (9.09)
Education level	
Bachelor’s degree	2 (10)
Master’s degree or equivalent	13 (65)
Doctoral degree or equivalent	5 (25)

1 Participants could self-identify with >1 race/ethnicity.

### Qualitative findings by SEM category

The recorded interviews lasted a mean of 36.3 min (range: 19.2–56.5 min). Thematic analysis resulted in a codebook with 6 themes and 27 subthemes. The first 2 themes (FIM defined and plant-rich diet defined) aligned with how participants defined these concepts, and the other 4 themes aligned with the 4 SEM levels (individual level, FIM implementing organizations, FIM community context, and policy environment). Figure 1 depicts these SEM levels along with the definition researchers developed to inform the bounds of each level. Supplemental File 2 lists the subthemes within each SEM level along with illustrative quotes for each subtheme. Given the interdependent nature of SEM levels, some participant observations were co-coded across different SEM levels. Although the following sections present participant quotes by SEM level, Supplemental File 2 provides examples of how participant reflections may have crossed SEM levels.

**FIGURE 1:**
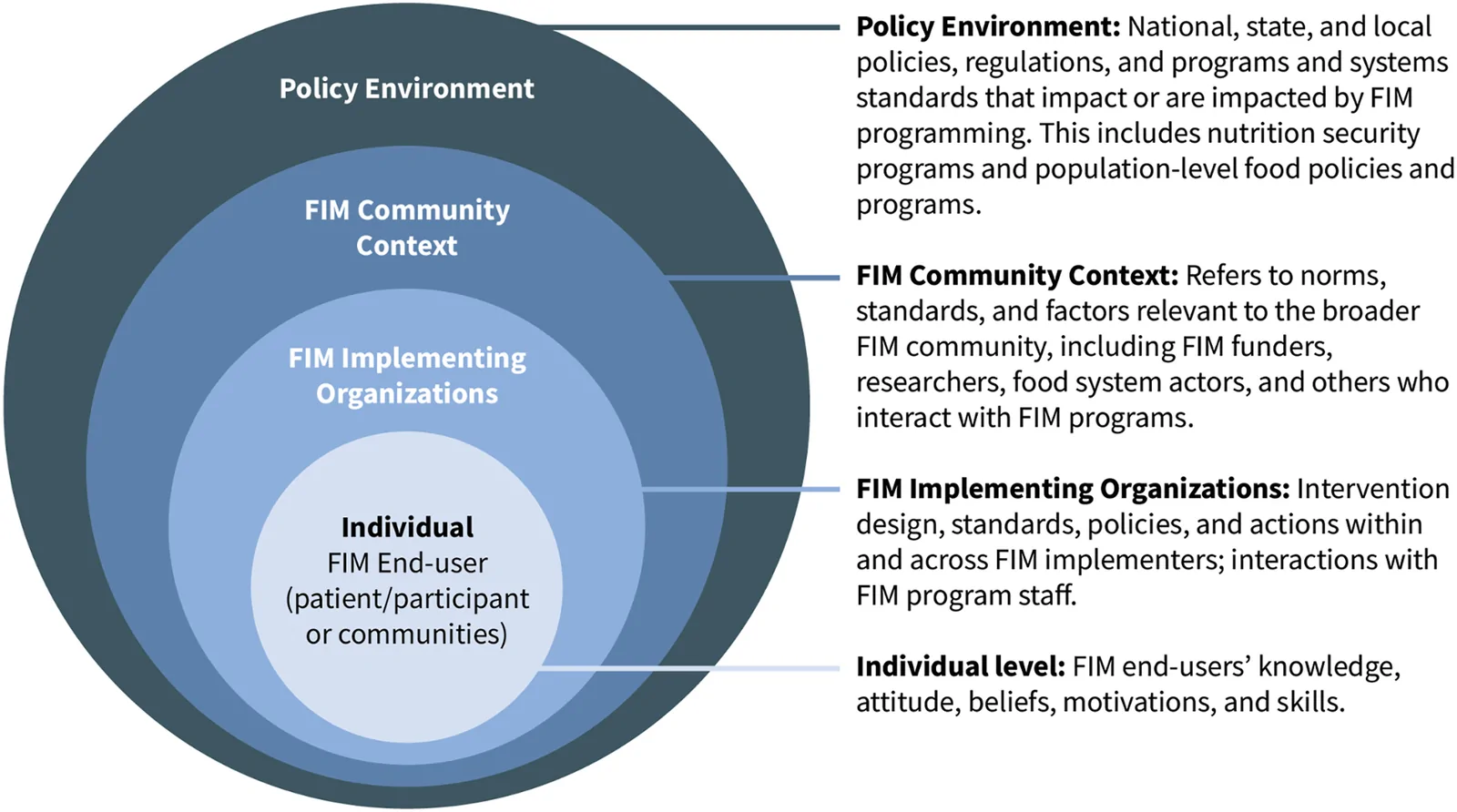
Socioecological model themes and definitions used for the thematic analysis of FIM programs. FIM, Food is Medicine.

### FIM defined

Participants reported that there was no universally agreed-upon definition for FIM. As a participant described: *“One of the issues in the field and with the public kind of perceiving what Food* *is Medicine means is that there's a broad spectrum of how people interpret that term. Everything from bioactive compounds and certain foods being ‘Food* *is Medicine,’ to views that we’re medicalizing a basic human need of nutrition”* (P3). Definitions varied between the experts interviewed for this study. Some participants described FIM from a public health lens, whereas others presented a narrower, clinical-focused view of FIM; only 1 participant (P1) mentioned sustainability within their FIM definition. Table 3 includes illustrative participant responses to reflect the variety of definitions provided.

**TABLE 3:** Illustrative participant definitions of Food is Medicine

Illustrative quote	Participant ID #
*“Using food and nutrition as a way to both prevent and treat both acute and chronic disease…. And so then, what I would add to it is, in a way that is affordable, accessible, delicious, and can be sustainable for farmers and the planet.”*	P1
*“…the integration of food and nutrition-type interventions or programs that are in close collaboration with the healthcare system, so it could either be in the healthcare system or like a partnership.”*	P7
*“…a dedicated, educated approach to eating and enjoying food, to help people live healthier lives, and to prevent illness before it starts.”*	P9
*“A framework that depicts a cascade of interventions that range from prevention-based programs to treatment-based programs in order to address food insecurity, diet quality, and diet-related chronic conditions concurrently. These programs are also used complementarily with nutrition education and counseling in some forms.”*	P14
*“…the utilization of food as a prescription for healthcare, in the same way that we use prescription drugs or surgical procedures.”*	P17

Multiple participants reflected that FIM is not a new concept, but one that has long been practiced by Indigenous communities and by those working with people with certain healthcare conditions (e.g., HIV/AIDS) and people with low income. Participants agreed that FIM represents a range of interventions or approaches. Yet, which interventions constitute FIM differed. A few participants saw the bottom 2 FIM Pyramid levels as complementary or adjacent to FIM but not FIM programs themselves. As one shared: *“I would say, the top three levels [of the FIM Pyramid] are really the core Food* *is Medicine interventions…the bottom two rows of the pyramid have, in the work I do, they’ve muddied the waters a little bit in—that’s my perspective—when we try to describe what Food* *is Medicine is. Because the bottom is everything we’ve been doing in public health nutrition, everything I’ve been working on my whole career”* (P13). Some participants considered the Special Supplemental Nutrition Program for Women, Infants, and Children (WIC) as an outlier compared with other long-standing public health nutrition programs, as it has been regarded as the “original FIM intervention.” A few participants noted that work is underway to create a standardized FIM definition. Some mentioned that to enact policy or legislative changes to fund FIM programs, such as covering MTM and MTG programs through Medicare, a narrower FIM definition is needed.

### Plant-rich dietary patterns defined

Participants broadly described that unprocessed or minimally processed plant-based food products should form the basis of a plant-rich dietary pattern. Some individuals shared that animal-based foods, particularly beef and other red meat sources, should be deemphasized, although not necessarily avoided entirely. Interviewees most frequently referenced the Mediterranean, vegetarian, and vegan diets as approaches they viewed as plant-rich and that were familiar to United States consumers. However, many participants noted that any population, community, or culture’s way of eating can have a plant-rich approach. For instance: *“…people get confused and think, ‘oh, you talk about a plant-rich diet being healthy for the planet and for people. That means vegan.’ And no, it doesn’t. It is what it says. It’s more plant-forward, it’s bringing that to life through the particular culture and community”* (P5).

### Individual FIM end users

Participants emphasized that FIM programs must meet end users where they are when it comes to food and cooking knowledge, access, and skills; individual and cultural preferences; and their willingness and interest to try new foods. As one shared, FIM programs should be *“designed hand-in-hand with community members”* (P3) to ensure they meet their needs. Relatedly, supporting patient/client choice and dignity was frequently discussed.

Multiple participants mentioned that, in general, United States consumer demand for products that are locally grown is increasing and that individuals, especially younger consumers, want to know where their food comes from and who supplies it. Another participant stressed that, beyond just providing food, FIM programs need to empower individuals and communities to *“be stewards of their own diet, health, and land and environment”* (P12).

Many participants noted the importance of incorporating education and culinary training into FIM programs. As one MTM implementer described, *“We think it’s really important to pair education along with our produce, so people can learn how to prepare it and use it and try it. Some people haven’t even tried these fruits and vegetables”* (P11). Education could help to better connect consumers to agricultural practices: *“So I think that starting with education, not just on the importance of plant-rich diets, but where do the plants come from, and how do we grow them?…I think that the time is right for that. Everybody is really very tuned into homesteading to gardens in the city, rooftop gardens. I think people are ripe for this kind of conversation”* (P6). Education can help with long-term behavior change. *“I do think it’s crucial to have nutrition education, and even for the medically tailored meals, I think it’s still crucial, because at some point, at least, right now, those are gonna end for that person. So how do they learn how to make that themselves? So that they’re still eating healthfully after whatever set period of time their insurance is gonna cover it…”* (P10). Yet, some participants shared that nutrition education varies across FIM programs, with some programs focusing on food access as the core issue, whereas others believe food provision should be paired with nutrition education.

### FIM implementing organizations

Many interviewees provided examples of how FIM programs are already supporting plant-rich dietary patterns. Participants identified produce prescription programs as supportive of plant-rich dietary patterns by default, because they focus on increasing fruit and vegetable intake. Similarly, many saw MTM and MTG programs as largely supportive of this approach. For instance, one participant shared: *“I think many programs that offer medically tailored meals are trying to make sustainable choices in the types of foods that they provide because many of those foods are really the types of foods that are important for an individual's health”* (P15).

Yet, participants shared that FIM programs widely differ in how they are designed, including the provision and intensity of nutrition education, the methods for food delivery and government benefits transfer, the level of consumer food choice, and the quality and types of foods provided. This presents challenges for cohesively promoting plant-rich dietary patterns because each implementer is doing things differently. The benefit of this diversity is that it allows flexibility to design interventions that meet the local community’s needs. However, participants raised concerns around the lack of standards and quality controls for some FIM programs. Participants mentioned how nonprofit MTM providers have accreditation standards for meal quality, developed by the FIM Coalition. Similarly, when FIM programs work with a registered dietitian nutritionist, the meals or education should be based on clinical guidelines used for Medical Nutrition Therapy. Yet, others shared concerns that some companies’ FIM products might not be considered healthy food options: *“You now have these food companies, mostly for-profit medically tailored meal providers like [MTM company], that are doing the Jimmy Dean-style sausages that are mailed to people’s doors, and other companies that are trying to sell you better-for-you granola bars and calling it Food* *is Medicine..*. *So we have to have standards to make sure that the food is healthy, and what does healthy mean?”* (P19). Participants shared that existing FIM standards do not consider environmental sustainability.

Participants described how some FIM pilot programs incorporate sustainability principles, such as reducing food waste and sourcing from local and/or regenerative agriculture producers. As one shared: *“I think there’s also interventions that are focused on things like regenerative soil and have a much greater focus on food systems resilience, so making sure that you’re supporting local and reducing food waste, which I think all ties into the sort of planetary health message, even though it may be not directly like speaking to planetary health in the messaging”* (P4). One participant spoke about produce prescription programs specifically explaining, *“I think there’s so many great models of local farmers and trying to drive to that local infrastructure as well”* (P15). They went on to explain how this local purchasing can also benefit farmers because, if FIM programs purchase in advance, *“it helps ensure that the farmer is gonna sell the produce that they’re growing”* (P15). Participants shared the need for more rigorous studies and data to support the effectiveness and impact of FIM programs to help with implementation and to guide FIM scale-up. This could include expanding the monitoring and evaluation metrics beyond human health to assess the planetary health and economic co-benefits of some FIM models.

Looking forward, many participants saw opportunities for better integrating sustainability considerations within FIM programs. Doing so across all aspects of FIM program design and implementation was viewed as necessary to be effective. As one expressed, *“…there is an opportunity, if they are prioritizing local sourcing and things like that, to be able to impact planetary health as well on like a larger level. If that was a priority across the board. I feel like that could be a strong opportunity if that was baked into the design of a lot of the programs”* (P7). Another emphasized: *“1115 Medicaid waivers unlocking dollars to serve population health using the Medicaid system is great, and we’ve got the time now, and perhaps only now to say, and here’s a corresponding set of frameworks around these 1115 dollars that would support more planetary health, by way of local supply chains, by way of sustainable practices, by way of organic or otherwise humane certifications”* (P18).

However, some participants shared that incorporating sustainability practices across all FIM programs may not be tenable. For instance, *“…funding levels are so low for these programs, so what’s realistic? And you may start putting so many constraints and restraints on the program that they’re untenable, so that’s the balance”* (P4). This view builds on existing FIM program challenges mentioned around food access, costs, and perishability; produce/meal uptake and consumption; technology; and logistics. Other participants expressed that supporting local economies and other sustainable diet practices are beyond the scope of FIM programs. As one shared, *“…We’re trying to make produce prescriptions as close as possible to any other clinical treatment.* *We think we are going beyond our bounds and asking too much of this clinical treatment to also help a local economy”* (P17).

### FIM community context

Multiple participants expressed that those in the FIM movement are *“riding a wave of momentum at this point that won’t necessarily be here in the future”* (P3) and emphasized concern around how to *“keep it [FIM] moving forward regardless of political climate and* *funding”* (P20). This FIM momentum was regarded as a major moment and opportunity for RDNs, who many participants described as integral to FIM programs—and for promoting plant-rich dietary patterns within these programs. Participants raised concerns about the declining RDN population and its effect on FIM scale-up, as *“all of this great stuff we’re doing can’t happen without an RD”* (P6). Similarly, participants shared a need for health care providers, especially physicians, to have more nutrition knowledge to educate their patients and refer eligible individuals to FIM programs and RDNs. When it comes to support sustainable diet practices, some interviewees mentioned how RDNs and others working within FIM could be better systems thinkers to assess the potential cobenefits of these programs more holistically. As one participant shared: *“I think sometimes a lot of these programs and the efforts for them do happen kind of in silos. And so, making sure to have coordinated efforts across, say, like government, researchers, practitioners to make sure that we’re really studying and understanding the impact of these programs at a large scale. So, I think that's the challenge is making sure that they’re just like coordinated efforts across the board”* (P7).

Participants described the need for systems thinking and integration across FIM for the health and food systems to engage with and learn from each other, moving away from the siloed approach to addressing health and agriculture challenges. One participant stated: *“An observation that I think many of us have is that the healthcare system is not the food system, and there’s a learning curve both for food system actors and activists to learn about the healthcare space, and, in reverse, healthcare folks learning about the food space”* (P18). This will require bringing diverse voices from health and agriculture spaces to the table. Similarly, many participants described how different messaging around the human health, economic, and planetary health benefits of FIM could attract diverse stakeholders. A few feared that describing FIM as beneficial for addressing climate change could discourage support from some policymakers and funders. One participant shared that they were *“actively shutting down that conversation in [Washington] D.C.”* because they *“believe that message hurts our policy chances for healthcare utilization uptake”* (P17).

### Policy environment for supporting FIM programs

Participants described many challenges to promoting sustainable diet practices within FIM programs and beyond because of existing federal policies and norms and a lack of political will to move this agenda forward. Many stated that receptivity to these concepts will depend on the values of the political administration. Moreover, participants mentioned that states in the United States can likely have more flexibility to advance a plant-rich diet agenda and incorporate these principles into FIM programs than the national level, which depends on Congressional support.

A few participants raised concerns around whether incorporating sustainability concepts into FIM programs would further politicize the discourse on FIM or cause people to disengage. As one participant shared, *“Food as Medicine or Food* *is Medicine is already complicated, and climate change is already complicated, so any communication bringing them together should be clear. My concern would be that, if at the beginning it seems too complicated, some people might not engage”* (P8). Yet, some also noted that growing FIM momentum offers an opportunity to bring food, health, and agricultural policymakers together to discuss larger structural changes needed to the food and health systems. *“Food too should be able to cross the aisles to health, and the only way to do that is setting up the infrastructure to where those individuals that have been on one side of the aisle come together. So, if we have it in the way we talk about colors to kids, red and blue over here make purple. Then Food* *is Medicine is the purple, and we need to bring those folks on the extreme sides of medicine and food and ag. to come and have a seat at the table, and that started with the [2022] White House Conference on Hunger, Nutrition, and Health”* (P16).

Some participants referenced the control of large food and beverage corporations and the meat and dairy industries over the food system and their policy influence as a main challenge to shifting the current food system model (and FIM) to better emphasize plant-rich dietary patterns. Existing federal food subsidies were mentioned as a barrier. *“We’ve got this recommended plate from the USDA [U.S. Department of Agriculture], where half of all the food [on the plate] we consume should be fruits and vegetables, and yet one-half of 1% of Farm Bill funding goes to supporting ‘specialty crops,’ aka food that humans eat. What about right-sizing that? And I know that's rife with all kinds of issues that are multi-generational at this point”* (P18). Many participants mentioned the need for the United States government to incentivize or create policies that require agri-food system stakeholders (i.e., manufacturers, growers, producers, companies, retailers) to make changes that support both human and planetary health. They also mentioned the need for the United States food and health systems to better support culturally diverse foods and ways of eating, such as the African heritage diet.

Additionally, participants offered existing actions and opportunities within federal policies and programs to support sustainable diet practices. Participants shared that the Supplemental Nutrition Assistance Program (SNAP) was not set up to promote plant-rich dietary patterns. Double-up Bucks and other programs that incentivize SNAP and WIC users to purchase more fruits and vegetables were described as existing initiatives that support plant-rich dietary patterns. Some interviewees observed a move toward plant-rich diets in WIC and school meals in certain districts and states, providing examples of schools that increased plant-rich offerings to students during the COVID-19 pandemic. Many participants described school meals as an outlet through which to educate children about plant-rich dietary patterns and planetary health to drive lasting behavior change.

In the absence of federal policy that connects food and health systems, multiple interviewees shared that a bottom-up approach to sustainable diet change is more likely to succeed. As one participant noted: *“I think there’s a real important absence of political will on this topic—that the generation of politics will begin with individual choices, and then extending those to families or local influencers and then moving out beyond institutions into local government and then state government, and those are the kinds of arenas that we’re working in”* (P2).

## Discussion

Findings from interviews with 20 FIM experts in the United States indicated that many current FIM actions support plant-rich dietary patterns and other sustainable diet practices, and that challenges and opportunities exist to further incorporate and standardize these principles across FIM programs. Although many of the participants we interviewed shared favorable sentiments around incorporating sustainable diet practices in FIM programs, as our results show, others were less optimistic about the potential alignment. We also note that some stakeholder groups (e.g., media, professional societies) were underrepresented in our sample, so their perspectives may not be fully reflected in the results and our interpretations of them.

In the following sections, we first reflect on participant-derived definitions for FIM and plant-rich dietary patterns. We then situate the interview findings within the context of their implications for policy (including current political tensions), research, and practice to describe how United States food and health system actors could incorporate sustainable diet considerations into FIM. The research and practice implications build on findings that spanned the individual, FIM implementing organization, and FIM community context levels of the SEM model, whereas the policy implications build on findings from the policy environment level.

### Guiding definitions

Participants in this study were not in agreement on what constitutes FIM, with some providing broad definitions that could encompass a variety of policies and programs and others focusing more narrowly on MTM, MTG, and produce prescriptions as FIM programs. Although the Department of Health and Human Services (HHS) has coordinated government-wide FIM framing language and principles [33], many participants did not reflect this language in their FIM definitions. Likewise, although the FIM Pyramid has been widely used within FIM research, several participants in this study believed that the FIM Pyramid was too broad to support FIM policy and advocacy goals. Conversely, participants shared similar definitions for and characteristics of plant-rich dietary patterns. These participant definitions largely aligned with definitions from national and global expert bodies, such as the Eat Lancet Commission, the World Health Organization (WHO), and the United Nations Food and Agriculture Organization—including the definitions that guided this research study [1,[8], [9], [10], [11]]. Future research could explore how to expand upon and revise the FIM Pyramid and prominent guidelines (e.g., the FIM Coalition MTM accreditation requirements) to incorporate how FIM programs can promote sustainable diets across multiple dimensions.

### Research and practice implications

Participants described a lack of standardization across FIM programs. This aligns with findings from other studies regarding varying design, implementation, and evaluation approaches for FIM programs given a lack of shared standards and best practices, making it challenging to conclude the overall effectiveness and potential for these interventions [[34], [35], [36]]. This lack of standardization could stem from the lack of a unified FIM definition as noted previously. Developing standardized definitions and evaluation metrics for FIM programs could help address these concerns and could be designed to encourage planetary health co-benefits. For instance, HHS has developed a FIM analytic framework with a domain focused on Food Quality and Production Effect [33]. Although deemed “noncore requirements” in FIM programs, the framework measures success in this domain based on increased regional food sourcing, strengthened local food economy, greater support for regional food producers, and transformation and expansion of local food systems [33]. This domain could be expanded upon to provide values-based implementation and evaluation metrics by which all FIM programs could track progress in this domain. The Good Food Purchasing Program has purchasing strategies and sourcing targets for food service institutions related to promoting local and community-based economies, environmental sustainability, valued workforce, animal welfare, and community health and nutrition [37] that could be adapted to help FIM programs track and share sustainable diet-relevant impacts.

Many interviewees expressed that FIM could be a pathway to promote plant-rich dietary patterns because these programs rely on plant-based foods. Interviewees described pilot FIM programs that have incorporated other sustainable diet practices, such as local food purchasing, regenerative agriculture practices, or food rescue efforts to support planetary health—similar to examples shared in Rahman et al. [24], Gupta et al. [38], and Ridberg et al. [39]. Yet, as participants mentioned, these practices are not universally incorporated into FIM programs, and FIM programs are not widely messaged for their planetary health cobenefits.

Participants also emphasized the key role of nutrition education in FIM programs, although whether or how programs implement this education is not standardized [40]. When implemented, nutrition education has been associated with greater improvement in FIM participant outcomes, including improved food and nutrition security [41]. Supporting FIM programs to implement nutrition education could therefore enhance health outcomes and promote sustainable diets as well. For instance, FIM implementers could use these educational opportunities to shift consumer behaviors toward plant-rich dietary patterns and practices that align with their individual and cultural preferences. Nutrition education can also help FIM participants learn how to properly prepare and store provided foods, reducing food waste [22].

Incorporating sustainability principles throughout FIM program design offers an important opportunity to elevate the interconnected nature of human and planetary health. A 2026 Rockefeller Report found that if United States FIM programs prioritize local food systems within program design, they can provide ≥$5.6 billion in new revenue for small and mid-sized farms, create and support jobs across the food value chain, enhance community resilience, and provide environmental benefits [42]. Rahman et al. [24] described how integrating local and sustainable sourcing requirements for Medicaid and Medicare reimbursements could support broader sustainable food sourcing within FIM programs. Future research could explore current sustainable diet-relevant metrics used in FIM pilot programs and adopt dissemination and implementation science applications to explore the barriers and facilitators to wide-scale integration of these practices across FIM programs [43,44]. Likewise, additional research is needed to quantify the potential environmental impacts of implementing sustainable diet practices within FIM programs, such as utilizing life-cycle analysis or environmental impact assessment measures [22].

Incorporating sustainable diet practices into FIM programs is not without its challenges. As participants shared, some FIM programs already face limited capacity and resources. Adding additional logistics, partnerships, and costs associated with values-based purchasing and comprehensive nutrition education may stretch program resources too thin. Sustainable diet principles and practices could therefore be prioritized during program design, be informed by and aligned with community needs, and be supported by program funders to minimize implementation barriers.

### Policy implications

Participants noted that sustainability or climate messaging may not resonate with certain policymakers. These interviews were conducted in the months before a change in administration. The current administration has taken steps to distance the United States from climate conversations and action, including withdrawing from the United Nations Paris Climate Agreement [45]. These changes may make some policymakers even less receptive to sustainability or climate-focused messaging than when we conducted these interviews. Participants also raised concerns around whether national FIM momentum was temporary and may shift with changing government administrations. Funding cuts to food and nutrition programs, such as SNAP, and to Medicaid in 2025 have likely hindered the pathways for some FIM programs [46,47]. At the same time, the Administration’s Make America Healthy Again (MAHA) movement has prioritized reducing diet-related chronic diseases and emphasizes the connection between food, nutrition, and health outcomes [48]—which could align with FIM objectives.

As participants described, many aspects of the United States food system, such as the agricultural subsidy structure, present barriers to implementing sustainability considerations. FIM alone is unlikely to right-size these system barriers. Momentum around MAHA and FIM could present an opportunity for food and health system stakeholders to use systems thinking to develop FIM programs with co-benefits for human and planetary health. Amid a challenging government funding environment, FIM implementers could also use sustainability messaging and program framing (e.g., prioritizing regenerative, organic, and local food farming and purchasing; plant-rich diet education; food waste reduction) to attract new and diverse climate-focused funders to collaborate on common goals.

### Strengths and limitations

To the authors’ knowledge, this is the first study to assess FIM stakeholders’ perspectives on the opportunities and challenges of incorporating sustainable diet principles and practices into FIM programs in the United States. Our use of stakeholder mapping to identify and categorize stakeholders was a strength as it encouraged a diversity of perspectives from individuals and groups working on and engaging with FIM in different ways. One researcher (KCS) conducted all interviews as part of her PhD dissertation. A brief reflexivity statement was therefore included in the methods section to help orient readers to potential researcher subjectivity. The use of a second researcher to independently code each interview aimed to limit researcher subjectivity in the coding process and achieve consensus, although subjectivity is inherent in qualitative analysis [25]. Consensus coding enhances reliability and can help encourage deeper discussion between coders on the themes emerging from the data [49], but alternative interpretations of the data may have been underexplored. To address these limitations, the researchers documented coding consensus decisions and used cocoding across different SEM levels to capture different meanings reflected within a single statement.

Although data saturation was assessed and achieved across groups, because some expert groups were more represented than others in this study (e.g., few industry representatives responded to interview requests), data saturation may not have been reached within each group. We relied on publicly available information to contact potential participants, so those without public e-mails or LinkedIn presence may be underrepresented in this study as we lacked means for contacting them. Snowball sampling may have limited diverse representation in the participant pool, as participants may have referred us to others with similar worldviews. However, a minority of our sample was identified through this snowball sampling approach. Two interviewees were from the same academic institution, which may have limited the diversity of perspectives in the sample.

In conclusion, findings from expert interviews indicate that FIM programs largely align with plant-rich dietary patterns. Some FIM pilot programs have incorporated other sustainability considerations, such as supporting local food systems and reducing food waste, but these practices are not standardized across FIM programs. Likewise, FIM programs are not widely messaged as supportive of planetary health. Unified FIM definitions and standards could help reduce variation across current FIM programs and could incorporate sustainability principles, systematically integrating these practices into FIM program design. Likewise, future research might collect sustainable diet-relevant implementation and evaluation metrics from across existing FIM pilot programs to assess the potential for these programs to provide planetary health cobenefits. Participants shared many opportunities for more holistically incorporating sustainable diet practices into FIM interventions, but limited FIM program bandwidth, an unsupportive political environment, and policy and systems barriers may inhibit the enabling environments for these opportunities. The momentum around the FIM and MAHA movements could be leveraged to enhance food and health system collaboration and develop FIM programs that address both human and planetary health.

## Author contributions

The authors’ responsibilities were as follows – KCS, VIK: designed the research; KCS: conducted the research and wrote the first draft of the manuscript; KCS, PBH, BH: analyzed data; PBH, BH, ES, AH, VEH, VIK: provided feedback on the interview guide and draft manuscript; KCS: took primary responsibility for final content; and all authors: read and approved the final manuscript.

## Data availability

The codebook used in this study will be made available on reasonable request to the corresponding author. The interview data analyzed for this study will not be made available to protect participant confidentiality.

## Declaration of generative AI and AI-assisted technologies in the writing process

The authors declare that no generative AI or AI-assisted technologies were used in the writing of this manuscript.

## Funding

VIK—partial funding for salary was supported by the Hatch project award VA-160189 from the USDA’s National Institute of Food and Agriculture.

## Conflict of interest

The authors report no conflicts of interest.
