# Supplementary material for: Opportunities and Challenges for Promoting Sustainable Diets within “Food is Medicine” Programs in the United States: A Qualitative Research Study
Source: Curr Dev Nutr. 2026 Sep 23;10(10):109541. doi: 10.1016/j.cdnut.2026.109541 (PMC13683016; doi:10.1016/j.cdnut.2026.109541)
Supplement: Supplementary file 1 [file mmc1.pdf]

Opportunities and Challenges for Promoting Sustainable Diets within "Food is Medicine" Programs in the United States: A Qualitative Research Study
Katherine Stanley, et al. 2026
Supplementary Files

Supplementary File 1: Semi-structured interview guide for expert interviews for promoting sustainable diets within Food is Medicine programs in the United States

This study seeks to assess whether and how plant-rich dietary patterns can be encouraged within U.S. Food is Medicine interventions to support human and planetary health. Thank you for agreeing to be interviewed for this research project.

The interview will last about 45 minutes. During the interview, I will ask you a series of questions about your views, opinions, and perceptions related to the role of plant-rich dietary patterns in Food is Medicine interventions. There are no correct or incorrect answers to any of the questions. My co-researchers and I want to hear your honest views and experiences. We will view the responses you provide in this interview as your own rather than those of the organizations or agencies with which you are affiliated. While we cannot offer any financial compensation for participating in this study, we are happy to share the final results with you once the study is completed.

A. <u>Informed Consent</u>

I would like to record the Zoom interview to reference the responses more easily in the future. The recorded interview will be kept confidential and not be shared beyond our small research team, and it will be contained within a password protected folder. The video will be labeled with your participant number rather than your name or any other personally identifiable information. However, if you do not feel comfortable with using video, or do not have stable internet to allow for video use, we can conduct the interview using audio only. In this case, we will record the audio. You will not be asked to provide your name or any other personal information, aside from the organization or entity for whom you work, while we are recording the interview, so your

information will not be linked to the audio or video file. Participants will only be classified based on the participant group(s) with which you align (e.g., government agency, civil society organization, academia, etc.) for all data analysis and coding. The results will be used to inform a PhD dissertation and incorporated into a manuscript for publication.

Your participation is voluntary. You have the right to end the interview at any time. If you do not feel comfortable answering a particular question, please let me know, and we will move on to the next question. Before we proceed, do you have any questions?

I would like to confirm that you give consent for me to record this video interview today?

B. <u>Interview</u>

I have started the recording. This is participant #xx who I am interviewing on *[month, day, and year]* at *[time]*.

Q1: How would you define or describe Food is Medicine?

Q2: Could you please briefly describe the scope of your work or that of your organization as it relates to the five levels of Food is Medicine prevention or treatment interventions in the U.S.? *[Slide shared with an image of the Food is Medicine Pyramid*]

Q3: How would you define or describe plant-rich dietary patterns?

Probing question: What specific dietary patterns in the U.S. meet these criteria?

I will now share the definitions that we have used to inform this research study. The World Health Organization and United Nations Food and Agricultural Organization define a sustainable diet as dietary patterns that support human health and well-being for current and future generations, minimize environmental impact, and are affordable, accessible, equitable, and culturally acceptable. Sustainable, plant-rich patterns have co-benefits for human and planetary health, which is defined as the health of human civilization and the natural systems on which humans depend. These co-benefits include reducing diet-related chronic disease risks, improving health outcomes, and reducing the food system's environmental or ecosystem burdens.

Next, we are going to discuss your views on the role of plant-rich dietary patterns within Food is Medicine interventions guided by your work within the five levels of the Food is Medicine Pyramid.

[Questions 4-7 were tailored based on participant's response to question 2, starting with the questions most relevant to their level(s) of work. Participants were then asked about their ability/interest in speaking to the other levels.]

Q4: What role do you believe that sustainable, plant-rich dietary patterns can or should have within **medically tailored meal or grocery programs** to support human and planetary health?

> **Probing questions:** What examples do you know of plant-rich dietary patterns incorporated into these meal or grocery programs? What challenges exist to incorporating plant-rich dietary patterns into these programs? What opportunities exist for doing so?

Q5: What role do you believe that sustainable, plant-rich dietary patterns can or should have in **produce prescription programs** to support human and planetary health?

Probing questions: E.g., opportunities through accompanying nutrition education alongside prescriptions themselves? Cooking demos? Standards for what should be included with this? What examples do you know of plant-rich dietary patterns incorporated into produce prescription programs? What challenges exist to incorporating plant-rich dietary patterns into these programs? What opportunities exist for doing so?

Q6: What role can or should sustainable, plant-rich dietary patterns have in **federal food and nutrition assistance and nutrition security programs**, such as the Supplemental Nutrition Assistance Program (SNAP), WIC, or school meals, to support human and planetary health?

Probing questions: What examples do you know of plant-rich dietary patterns incorporated into these programs? What challenges exist to incorporating plant-rich dietary patterns into these programs? What opportunities exist for doing so?

Q7: What role, if any, do you believe that these patterns can or should have in **population-level healthy food policies and programs** to simultaneously support people's personal health and planetary health?

Probing questions: What examples do you know of that promote plant-rich dietary patterns for human and planetary health? What challenges exist to incorporating plant-rich dietary patterns into population-level policies and programs? What opportunities exist for doing so?

Q8: Aside from promoting plant-rich dietary patterns, are there other sustainability practices or actions that you think should be incorporated into the idea of Food is Medicine?

Q9: What additional policies or actions do you believe are needed for diverse food and health system actors to incorporate sustainable, plant-rich dietary patterns into Food is Medicine prevention and treatment interventions to align with or achieve the recommendations in the Biden-Harris Administration National Strategy on Hunger, Nutrition, and Health by 2030?

> **Probing questions:** What actors must be involved in developing and implementing these policies or actions? In your opinion, why have these policies or actions not yet occurred? What do you perceive as the challenges to implementing these policies or actions?

Q10: Aside from promoting plant-rich dietary patterns, are there other sustainability practices or actions that you think are or could be incorporated into Food is Medicine to support planetary health?

Q11: In your opinion, what might be the benefits to positioning Food is Medicine as an approach that supports planetary health in addition to human health – bring food sector more into it?

> **Probing questions:** What political or logistical reasons may prevent tying this initiative to planetary health? What might be the political implications if the Administration (i.e., White House and federal government) was to integrate sustainable diets into the Food is Medicine strategy?

Q12: What challenges or drawbacks might be encountered in positioning Food is Medicine as an approach that supports both human and planetary health?

Thank you for taking the time to speak with me and to share your perspectives on this issue. Is there anything else you'd like to share?

Supplementary File 2: Coding Themes, Sub-Themes, and Illustrative Quotes

**Theme**	**Sub-theme**	**Example Quote**	**Quote Co-coding** (if relevant)
FIM defined	Disagreement on bottom two levels of FIM pyramid	"So I think WIC is like a good example of a Food is Medicine intervention because it is for a particular health population. But I think SNAP and school lunch, I don't see them as Food is Medicine programs, though I do see them as those critically important adjacent systems, because when patients are coming into the clinic, and they're screened, they're food insecure, and if they're not enrolled in SNAP. So having those direct relationships to be able to refer and help enroll a patient is really important. But they're not designed with a health component. It's an economic enhancement that helps people purchase food, which is critically important. But it is not Food is Medicine, which is supposed to have a particular health outcome." (P15)	N/A
	Stakeholders define what constitutes FIM differently	"So, I think that Food is Medicine is the intentional use of conventional food or ingredients, either alone or as a complement, to maybe more traditional approaches to medicine or other approaches to managing health or attaining desirable health outcomes." (P8)	N/A
Plant-rich diet defined	Diversity of diets or ways of eating can be plant-rich	"Well, I believe, because the evidence base is really showing that plant-rich diets are supporting of more sustainable and healthy meal patterns for people and the planet. I think there's always an opportunity to look at it through the lens of whatever population, group and cultural and community representation. So, while we know that plant-rich is important for health and for sustainability, it also needs to come to life in ways that are supportive of a particular culture or community, with preferences, with celebrations, with everything that comes as part of that. I just state that in the fact that that's a difference where some people get confused and think, 'oh, you talk about a plant-rich diet being healthy for the planet and for people. That means vegan.' And no, it doesn't. It is what it says. It's more plant-forward, it's bringing that to life through the particular culture and community." (P5)	Meet end-users where they are

	Mediterranean, vegetarian, and vegan as predominant plant-rich diets	"Yeah, I think there's a couple that the Dietary Guidelines Advisory Committee is looking at a few that are more kind of plant-focused like a vegetarian dietary pattern. And then, just more plant-based in general, even if it's not vegetarian. I don't know actual names of them. I mean, there's Mediterranean, which is talked about a lot. I don't think that's really followed as much here in the U.S., so yeah, I don't know the name of one in the U.S. that people are largely following, but I think there's certainly a couple." (P10)	N/A
	Plant-rich dietary pattern definitions align with guidance	"Plant-rich dietary patterns are those that, you know, put fruits and vegetables and legumes and healthy grains sort of at the center of your plate and use animal products as, sort of a flavoring or more of a condiment, rather than the center of your plate." (P1)	N/A
Individual level	Consumer sustainability interest and knowledge	"...So I think that starting with education, not just on the importance of plant-rich diets, but where do the plants come from, and how do we grow them? I think that right now, people are very…I think that the time is right for that. Everybody is really very tuned into homesteading to gardens in the city, rooftop gardens. I think people are ripe for this kind of conversation. So I think connecting to agriculture and health is really important." (P6)	Plant-rich FIM potential
	Meet end-users where they are	"So we're pairing education with produce so that people know how to use it and store it. And they're not just given a box of produce, and they're like, 'Here you go. Here's like 2 eggplants. Have fun.' They're like, 'what is this? We don't even know.' We think it's really important to pair education along with our produce, so people can learn how to prepare it and use it and try it. Some people haven't even tried these fruits and vegetables." (P11)	Diversity in FIM program development, implementation, and education
FIM implementing organizations	Diversity in FIM program development, implementation, and education	"Yeah, I, so I think this is another area where it also really varies because some produce prescriptions, and I think Food is Medicine programs in general, it depends on sort of what the target population is, for lack of a better word. So, for example, there's lots of programs that are really specifically serving the needs of like a diabetic population or a pre-diabetic population. There's other programs that serve pregnant people. And so sometimes the nutrition education is really tailored to that specific community." (P4)	N/A

	Implementation challenges	"Yeah, I mean, the number one thing is cost. So, it's local foods and sustainably grown especially, that takes these really more intensive practices. It's a change. And so sometimes that upfront cost is expensive, and if they can get it cheaper, when a lot of purchasers are looking at bottom line, if there's a difference in cost, where do you add that value cost in there? That's the number one challenge I always hear from programs." (P15)	Local food systems
	Improved production and/or waste practices	"And I think there's also interventions that are focused on things like regenerative soil and have a much greater focus on sort of like food systems resilience, so making sure that you're support supporting local and that kind of reducing food waste, which I think all ties into the sort of planetary health message, even though it may be not directly like speaking to planetary health in sort of the messaging." (P4)	Plant-rich FIM interventions; Diversity in FIM development, implementation, and education (part of larger co-coded segment)
	Plant-rich FIM interventions	"So what's the original question? Okay, yes, the interventions. So I think that most medically tailored meals are gonna be plant-rich by default. They're going to be heavy in fruits and vegetables, legumes, beans, nuts, seeds. And then produce prescriptions just by default their produce. I think the debate there is sometimes, can we do frozen? Can we do canned? Is it just fresh?" (P13)	N/A
	Plant-rich FIM potential	"I think, as we look at things like 1115 Medicaid waivers. This is like back to the architecture thing as a verb—1115 Medicaid waivers unlocking dollars to serve population health using the Medicaid system is great, and we've got the time now, and perhaps only now to say, and here's a corresponding set of frameworks around these 1115 dollars that would support more planetary health, by way of local supply chains, by way of sustainable practices, by way of organic or otherwise humane certifications. You could do it a lot of different ways." (P18)	N/A
FIM Community Context	FIM scale-up and research	"So a lot of unanswered questions. And I think maybe somewhere there's planetary health, sort of putting a lot of eggs in one basket and nothing has ever done that. And also that doesn't mean that diminishes the potential and	Plant-rich FIM potential

		benefit of Food is Medicine. Because even with incremental change, if we can show that these interventions are cost-effective and improve health outcomes, that's good enough. So you don't necessarily need the cure. We need a lot of other factors to get to a cure. But incremental change is a good thing." (P19)	
	Health provider nutrition education	"So I mean, I think in general, it's no news that most physicians are poorly trained in nutrition. I think there's a lot of barriers, which are probably listed out somewhere, of why physicians would not be more engaged, everything from training, from their cultural sensitivity, to lack of time. But on the flip side, I think I've heard, you know, from people like [researcher], that physicians, especially residents, feel empowered by having something positive to offer patients. That it's personally troubling to them to send folks who have a chronic disease condition out into a community where they know that they're not going to be able to feed themselves well. So it often, I think, is something that they can feel really positive about when they participate in the programs." (P3)	N/A
	Local food systems	"And the planetary health aspect. Again, I don't have as much to draw from, although I would say that as a field, I believe that there is a priority for local sourcing for the produce. So again, with produce prescriptions, this prescription is fulfilled at food retail so that could be a grocery store but also could be a farmer's market. There are models that are more like a CSA box where you receive a box of produce. So again, I think local sourcing is a priority. While sometimes, because of the nature and the community that the program is in, some of that may not be like full-fledged if there's a certain grocery store that is closest that may be the one that the program is partnered with. However, also, I think not as much in our current programming, but there are programs that I know of that do go even further, and they look at more regenerative agriculture as another step toward being more sustainable and looking at kind of planetary health impacts as well for these programs." (P7)	Plant-rich FIM interventions
	Messaging for different audiences	"I think sometimes so like, for example, there's a Farm Bill, right? And if we want the Farm Bill to invest in Food is Medicine. You've got to make a case for why this fits in with the USDA's mission, and USDA's mission is	N/A

		to drive the agriculture economy of the country, and so if I go in and saying, produce prescriptions are great for health, to the Agriculture Committee or the USDA, they're going to be like, yeah, but how does it help ag systems? And that's when you need to make that case. Same with philanthropy who wants to invest in the climate as a crisis. This would be a good way to do so, and I need to make that case for them if they're going to invest in Food is Medicine programs. But if I'm talking to a doctor, yes, in theory, the quality of the air and water is important to somebody's health, but the doctor wants to know is, is this going to change the blood sugar in the patient? And so it's about audience and about what you're asking of them. And certainly there are ways that making that case is useful. But it depends on the audience, and it depends on what we're asking of them, because it's not necessarily helpful for everybody to hear that." (P17)	
	Systems thinking & integration	"I think the challenges are that systems change is hard. A) it's hard and complicated. B) it's big and interconnected. We're not talking about changing the food system. We're talking about changing the food system, the healthcare system, the political system, the economic system, our education system. It's all interconnected, which makes it harder still, and it is hard because there's a lot of power and money that's entrenched in the long-term design of how we got to where we are, and hoping that it stays roughly, kind of the same as what it is because it's working for those folks in those positions. So, it's a big uphill battle, and that is definitely going to be the challenge." (P18)	N/A
	RDNs as plant-rich spokespeople in FIM programs	"I think there's lots of opportunity, I mean, I think, especially when dietitians are involved in creating the meal plans and doing things. They're automatically gonna know that. Okay, like, most people generally are not eating enough fruits, vegetables, nuts, seeds, beans, and I would think that would be at the top of their mind if they're thinking about what foods they want to point people toward and promote." (P13)	N/A
	Temporary momentum	" I think momentum. I mean, it's like keeping it top of mind and keeping the interest and the funding. All those things are very important. And we're all kind of afraid about the next administration, what happens if something changes and, okay, there goes all our support for produce prescription	Political nature of plant-rich diets and planetary health

		programming and Food is Medicine, all this stuff. Anyways, I don’t wanna think about that..." (P20)	(part of larger co-coded segment)
Policy Environment	Big corporation control of food environment	"Moving forward from a sustainability of large retailers and large manufacturers, whether we're talking about the retail end or manufacturers on the consumer product good side, so the CPG side, needs to be able to produce things at scale. And then there needs to be economies of scale that create an opportunity for the business model to work. So there has to be a healthy balance of consumer desire, consumer want, consumer need. And then, at the same time, being good stewards of the environment. I think that there can be a good balance of that." (P9)	Improved production and/or waste practices; Consumer sustainability interest and knowledge
	Bottom-up approach to influence policy	"I think there's a real important absence of political will on this topic—that the generation of political will begins with individual choices, I think, and then extending those to families or local influencers and then moving out beyond institutions into local government and then state government, and those are the kinds of arenas that we're working in." (P2)	N/A
	Changing food environment for culturally-diverse diets	"So let's just take USDA and HHS. They do these cooperative agreements when it comes to research beyond the dietary guidelines, and so they determine what investments they will make in certain research. And so, as it stands right now, African diet, Caribbean, whatever you want to call the African diaspora food ways, that has not been ever on the agenda and investment. That needs to change. So whatever the pathway to get there, that's where it starts. And that means educating, not only those within the bureaucracy, so there's levels to this right? So it's educating the public." (P16)	N/A
	Communication lacking on plant-rich foods and climate effects of food system	"But I think the main thing is more research and more demonstrations of the value, not only to the benefits that providers feel in being able to offer these types of programs to their patients. The benefits to the patients, both their physical and their mental health, and then how we can really ascertain some of those benefits that are outside of the healthcare system. So benefits to planet health, benefits to local farmers, economic infrastructure and community engagement, and many of the other things that we see in small	FIM scale-up and research

		scale programs. But I think being able to showcase that on a larger scale will really be critical to uptake of it more kind of globally." (P15)	
	Dietary Guidelines for Americans (DGAs)	"And then, of course, our dietary guidelines are missing sustainability from so much of what they say, and those dietary guidelines are the basis for what our nutrition recommendations are, what our dietetics curriculum is, what is in a school lunch program. So there's a huge miss there." (P12)	N/A
	Federal food purchasing and service standards	"So we wrote the Food Service Standards for Federal Facilities, which is another activity that we promote through our [program recipients], and we have standards within there which help guide folks towards using more plant-based proteins in their venue planning for food service. We stop short of recommending, you know, vegetarian diets and so forth because our North star is always the Dietary Guidelines for Americans. So animal-based proteins are not excluded in the Dietary Guidelines. So yeah, we'll always fall on the side of the plant-rich, but not exclusively plant-based, diet." (P3)	Plant-rich dietary pattern definitions align with guidance
	Incentives or policies to guide sustainability	"Yeah. So, I think it'd be a combination, like I just mentioned. How do you create the incentive for the manufacturers, the growers, the producers of these foods with a sustainability focus as a priority? Again, it would be through legislation or regulation and incentives. That's how I believe that that would work." (P9)	N/A
	Plant-rich dietary patterns within federal programs	"Yeah, no. I think, within the schools, there are some districts that have done really cool things when universal free meal coverage kicked in, and then they had access to more funds, and so they were able to procure plant-rich items that kids were acceptable….So I think there's opportunities on that front for some creativity, again hitting the budgets hard." (P8)	N/A
	Political nature of plant-rich diets and planetary health	"Yeah, I think, it's a huge challenge. I guess, at the same time, there's momentum around Food is Medicine, so there might be a real opportunity to advocate for improving the quality of these programs at an early stage, especially if the recommendations are meant to help the public, help those that the programs intend to serve. But I don't think energy alone or opportunity alone is gonna move the needle. think this is probably a time to continue relying on the evidence, continue collecting data and making sure that we have as many rigorous studies that could inform the implementation of these programs to reach as many people that could benefit from it. But	N/A

		from an advocacy standpoint alone, I think it's fairly challenging to try to blend in plant-based dietary recommendations with Food is Medicine from what I see it." (P14)	
